# Natural Language Search Interfaces: Health Data Needs Single-Field Variable Search

**DOI:** 10.2196/jmir.4912

**Published:** 2016-01-14

**Authors:** Caroline Jay, Simon Harper, Ian Dunlop, Sam Smith, Shoaib Sufi, Carole Goble, Iain Buchan

**Affiliations:** ^1^ Information Management Group School of Computer Science University of Manchester Manchester United Kingdom; ^2^ Centre for Health Informatics Institute of Population Health University of Manchester Manchester United Kingdom

**Keywords:** searching behavior, search engine, research data archives, user-computer interface

## Abstract

**Background:**

Data discovery, particularly the discovery of key variables and their inter-relationships, is key to secondary data analysis, and in-turn, the evolving field of data science. Interface designers have presumed that their users are domain experts, and so they have provided complex interfaces to support these “experts.” Such interfaces hark back to a time when searches needed to be accurate first time as there was a high computational cost associated with each search. Our work is part of a governmental research initiative between the medical and social research funding bodies to improve the use of social data in medical research.

**Objective:**

The cross-disciplinary nature of data science can make no assumptions regarding the domain expertise of a particular scientist, whose interests may intersect multiple domains. Here we consider the common requirement for scientists to seek archived data for secondary analysis. This has more in common with search needs of the “Google generation” than with their single-domain, single-tool forebears. Our study compares a Google-like interface with traditional ways of searching for noncomplex health data in a data archive.

**Methods:**

Two user interfaces are evaluated for the same set of tasks in extracting data from surveys stored in the UK Data Archive (UKDA). One interface, Web search, is “Google-like,” enabling users to browse, search for, and view metadata about study variables, whereas the other, traditional search, has standard multioption user interface.

**Results:**

Using a comprehensive set of tasks with 20 volunteers, we found that the Web search interface met data discovery needs and expectations better than the traditional search. A task × interface repeated measures analysis showed a main effect indicating that answers found through the Web search interface were more likely to be correct (*F*_1,19_=37.3, *P*<.001), with a main effect of task (*F*_3,57_=6.3, *P*<.001). Further, participants completed the task significantly faster using the Web search interface (*F*_1,19_=18.0, *P*<.001). There was also a main effect of task (*F*_2,38_=4.1, *P*=.025, Greenhouse-Geisser correction applied). Overall, participants were asked to rate learnability, ease of use, and satisfaction. Paired mean comparisons showed that the Web search interface received significantly higher ratings than the traditional search interface for learnability (*P*=.002, 95% CI [0.6-2.4]), ease of use (*P*<.001, 95% CI [1.2-3.2]), and satisfaction (*P*<.001, 95% CI [1.8-3.5]). The results show superior cross-domain usability of Web search, which is consistent with its general familiarity and with enabling queries to be refined as the search proceeds, which treats serendipity as part of the refinement.

**Conclusions:**

The results provide clear evidence that data science should adopt single-field natural language search interfaces for variable search supporting in particular: query reformulation; data browsing; faceted search; surrogates; relevance feedback; summarization, analytics, and visual presentation.

## Introduction

Data science spans many domains of application. For the health care and health sciences domains, it has the potential to bring researchers into “just-in-time collaboration” over shared data and data behaviors. The “big data” or “broad data” of data science lends itself naturally to secondary data analysis (using existing data to answer new research questions), which is traditionally associated with research data archives. Indeed, this data reuse is critical for both application and data mashups and acknowledges the cross-disciplinarity of the gathered data and their importance in combinatorial use. Using archived data such as annual health surveys, data discovery, particularly the discovery of key variables and their inter-relationships, is important for analyzing data and interpreting results properly. In addition, data science makes no assumptions concerning the domain expertise of a particular scientist, whose interests may intersect many domains and thereby enrich the research.

Secondary data analysis has a number of key functions [1] in relation to data science: it allows researchers to link datasets to answer questions that the files could not address adequately in isolation [2]; it creates opportunities to explore associations between factors that were not anticipated at the time of data collection [3]; and it has value from an ethical perspective by increasing the potential benefits to society arising from public investment in the collection of the original data [1]. Although secondary data analysis is essential to many areas of science and policy research, it is often impeded by difficulties in data discovery; besides, finding the most appropriate data to use for analysis can be problematic. Typically, the researcher needs to find a handful of appropriate variables among collections of thousands, often spread across multiple datasets such as successive years of a repeated survey. Current data archive information systems do not optimally support this search process; indeed, they make a presumption that their users are experts within the domain, and therefore, provide complex advanced interfaces to support these “experts.” These interfaces hark back to a time when searches needed to be accurate first time as there was a high computational cost associated with each search. In this case, data scientists share more in common with the “Google generation” than with their single-domain, single-tool forebears.

Anecdotally, although following a Web search interface design (best expressed by “Google,” “Bing,” “Ask,” etc.) would seem like the best practice, there is little empirical evidence to support such a claim. While the need to improve access to data for research purposes is recognized [4], no studies to date have directly examined how the user interface of tools providing access to archives impacts on the researcher’s ability to discover and extract relevant data. Here, we report the results of a study conducted in collaboration with the UK Data Archive (UKDA) [5], the largest collection of digital research data in the social sciences and humanities in the UK. At the time of the study, access to data stored in the UKDA—including government and other large-scale surveys —was formally provided by the Economic and Social Data Service (ESDS [6]), a data archiving and dissemination service supporting the secondary use of data in both research and teaching. ESDS provided access to a wealth of data and had more than 250 research institutions registered to use its services.

Searching and accessing data from the UKDA has not been easy for two main reasons [7]. First, researchers had to work out which of the more than 5000 datasets stored in the UKDA could most appropriately be used to answer their research question. Getting a sufficient overview of what was available in each set was difficult, and researchers often picked certain datasets simply because they were familiar with them [8]. Identifying the appropriate variables within a dataset was a second problem. Surveys typically contain hundreds, if not thousands of variables (the Health Survey for England 2007, for example, contains more than 2000), and variable labels may not obviously reflect their content. To accurately identify variables of interest, the researcher must read the original questionnaire alongside supporting documentation, a process that can take days or weeks of work, and which may ultimately be fruitless: until the researcher has completed the process they do not necessarily know whether the dataset can answer their research question. Although both fully understanding a dataset and reading its documentation are important to the research process, it would save researchers a great deal of time if they could limit this in-depth exploration to datasets that were likely to be useful to them. Understanding other aspects of data use, such as how derived variables have been constructed, or how data from a number of years can be compared, is also problematic.

Current systems can be thought of as divided into two categories: (1) those that use a traditional advanced search interface [9], which expects accurate queries, patient users, and moderated and homogeneous data; and (2) those that use a Web search interface, which expects vague queries, impatient users, and an enormous and rapidly expanding collection of unmoderated and heterogeneous data [10]. We suggest that variable search for secondary analysis has more in common with the hostile search environment of the modern Web than it does with traditional search.

In this study, we compare two interfaces: one based on a “Google-like” Web search interface that enables users to browse, search for, and view metadata for individual factors and variables; the other a traditional “advanced” search user interface (which presumes the user knows what they are looking for). Although more data archives do now have this kind of interface, our study is important because there is very little empirical work in this area.

Our hypothesis is that variables will be easier to find in research data archives via a single-field natural language search interface, conforming to Marchionini’s Human-Computer Information Retrieval (HCIR) framework [11] and in particular supporting query reformulation; data browsing; faceted search; surrogates; relevance feedback; summarization, analytics, and visual presentation.

### Background

There are numerous websites that provide access to the results of large-scale surveys (eg, the Office for National Statistics [12] in the UK, Eurostat [13] in the European Union, and the Bureau of Labor Statistics [14] and Inter-University Consortium for Political and Social Research (IPCSR) [15] in the United States). Until recently, the majority of survey repositories primarily used traditional search for the discovery of entire datasets, although the inclusion of Web search interfaces for variable data discovery is becoming more common. Both the IPCSR website and the Rand Survey Metadata Repository [16] provide access to a number of quantitative surveys conducted around the world and offer a facility for searching datasets at the level of variables. As detailed in the “Study Impact” section, following this study the UKDA now also supports variable data discovery using a Web search interface.

### Traditional Search Interfaces

Traditional “advanced” search, and the interfaces that facilitate it, is based on a number of long-held premises. The most noteworthy in this context are the presumptions that the interface can expect accurate queries, that users are patient, and that the data will be moderated and homogeneous [9]. In some cases, especially within the scientific research domain, these assumptions hold true. In other cases, however, they do not reflect reality. This seems especially to be the case with regard to searches of variable datasets that seem to have more in common with the heterogeneity of the open Web. Increasingly, traditional search interfaces focused on delivering well-curated datasets (often already known to the user) are now looking for novel ways to fill the user expectation gap [17]. These systems are increasingly recognizing that providing access to relevant information adapted to the needs and the context of the user is a real challenge [18] and that contextual results are becoming more important. Furthermore, evidence suggests that the traditional search model predicated on users searching for particular information, the so-called information need, may not be as important as navigational searches [19]. Indeed, understanding the underlying goals of user searches is becoming increasingly important; for example, the previously unexplored “resource-seeking” [20] goal may account for a larger fraction of Web searches than previously thought.

Traditional search expects the user to have well-defined boundaries for the information they seek, along with a good knowledge of the terms and meta-data that may be used to describe that information. This is increasingly not the case, especially in the context of variable data discovery and user-centered approaches [21] so common in the broad domain of data science.

### Web Search Interfaces

Web search, and its offshoot of HCIR, recognize the deficiencies in the traditional search model, and thus expects vague queries, impatient users, and an enormous and rapidly expanding collection of unmoderated and heterogeneous data [10]. Indeed, the model of traditional search is changing, with the widespread use of Web search engines, employment of simple queries, and decreased viewing of results pages—changes that have resulted from algorithmic enhancements by Web search engine companies [22]. Large providers, such as Google, run around 10,000 experiments each year in an attempt to refine both the search engine and the search interface and interactivity [23]. We could conclude that the high level of experimentation makes Web search engines de facto best practice for all other search instruments with traditional interfaces not being able to match Google’s ability to adapt and refine their algorithms and interactions. This is a trend we can see in search result clustering [24] for instance.

It is therefore not surprising that about 85% of Internet users surveyed claim to use search engines and search services to find specific information [25]. These users have expectations that bleed from Web search into all other areas that require search. To a naïve user, all search activity is the same [18]. In this case, we suggest that variable search for secondary analysis has more in common with the extremely hostile search environment of the modern Web than it does with traditional search.

### Faceted Search and the Google Generation

The move from traditional search to Web search may be a result of changes in user attitudes and needs. The “Google generation” appears to behave very differently to older generations [26]. They are less confident about their searching prowess, demonstrated by the fact that they viewed fewer pages, visited fewer domains, and undertook fewer searches than older users [27]. In addition, tellingly, their search statements were much more the product of cut and paste. These characteristics—of relying less on working memory and demonstrating lower competence at multitasking—has knock-on implications for researching in an online environment [26,28]. To overcome some of these limitations, we have seen a rise in faceted search, which combines query and browse strategies and interactively provides an iterative way to refine search results [29]. Faceted search allows users to start very generally and then iteratively refine their searches by allowing them to apply multiple filters selectively. These filters can be based on taxonomies [30], simple classifications systems [29], or other spatial locations [31]—in some cases, they are generated from search results sharing some common overlap [30]. This faceted approach dovetails into the evolving behaviors of the Google generation, and assists in complex decision making [32].

### Beyond Web Search

For reasons ranging from an obligation to curiosity, Web search is now moving beyond the individual and into the social domain [33]. Users have a strong inclination to seek information from others during the search process. Indeed, search systems using statistical analytics over traces left behind by others can help support the search experience [34]. Furthermore, result clustering based on social networks in a crowdsourcing role [35] and grouped clusters displaying multiple tabbed search results [36] are also being increasingly used. These advances suggest a social component to dataset and variable retrieval will, in the future, be expected.

### Context of This Study

Access to the UKDA via ESDS was set up primarily to facilitate the discovery and download of entire datasets, and as such shares much with traditional advanced search interfaces. The system provided several ways in which users could access individual variable descriptions, including a dedicated variable search facility, but anecdotal evidence indicated that these were difficult to use and not an adequate substitute for reading the complete survey documentation. Recognizing these issues, the UKDA decided to work with the University of Manchester (UK) to develop a Web search interface [37] as part of the Economic and Social Research Council-funded Obesity eLab project [38].

This interface was designed to simplify the process of accessing survey data, by enabling people to look for variables of interest through a familiar, and potentially more suitable, interface. Researchers typed a query into a single search box and then browsed relevant results. Variables were displayed in a tabular format, with the description shown prominently, allowing users to see at a glance whether the variable was relevant to their research question.

Although there is a large body of research examining search behavior in information retrieval [39,40], there is little that directly examines, from a user’s perspective, how best to retrieve variable data from archived surveys. Our solution, called “MethodBox,” initially emerged from the need to understand HCIR as it related to variable data discovery. A requirements analysis was conducted to understand the difficulties users experienced with the existing traditional search interface to UKDA, and to pinpoint new features that would help users to identify variables and datasets that could be used to answer research questions. The MethodBox Web search user interface was then designed to make the search process as straightforward as possible for novice users, reflecting the fact that most of their information retrieval experience will have come from the Web [26,41].

### Current Traditional Search Interface

At the time of the study, access to data stored in the UKDA, including government and other large-scale surveys, was formally provided through a website hosted by the ESDS, which provided numerous facilities for searching the UKDA catalogues.

**Figure 1:**
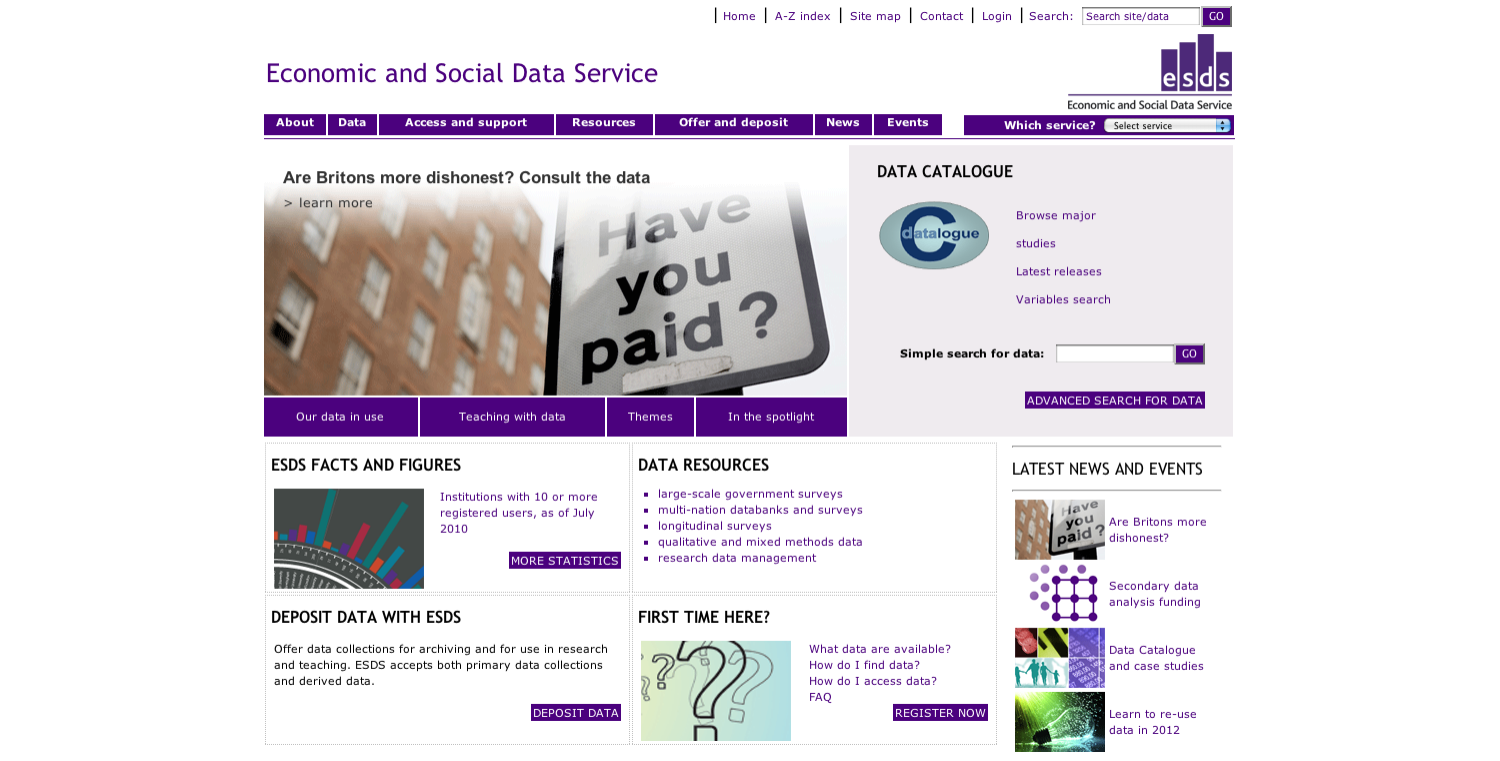
Simple Search -- Can be seen on the right-hand side.

On the home page (Figure 1), the simple search allowed users to search all fields in a record for keywords or phrases.

**Figure 2:**
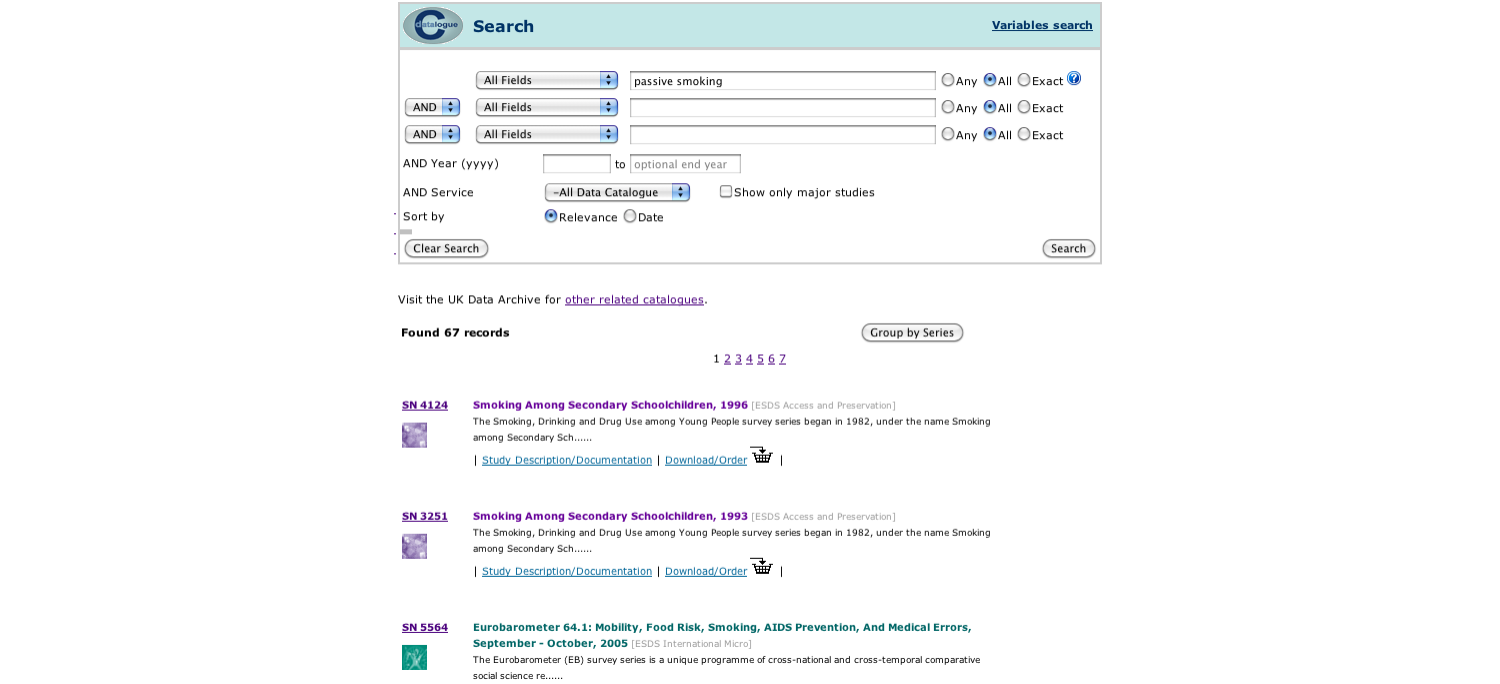
Orientation -- The search form is at the top of the page and the results are returned underneath. To view the variables, the user must click the 'Study Description/Documentation', then use the 'Variable List' link at the top of the page.

**Figure 3:**
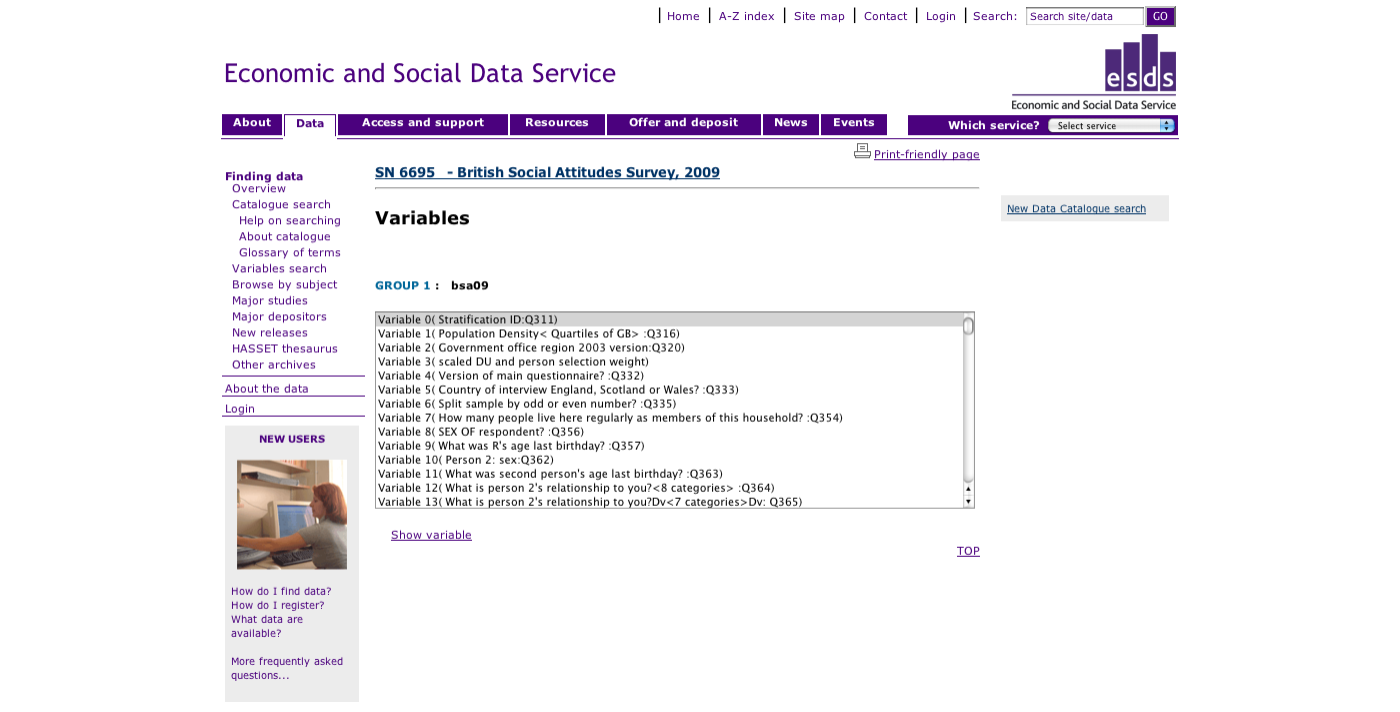
BSAS 2009 -- The variable list in the British Social Attitudes Survey 2009. To view a variable, the user selects one from the list box and clicks 'show variable’.

**Figure 4:**
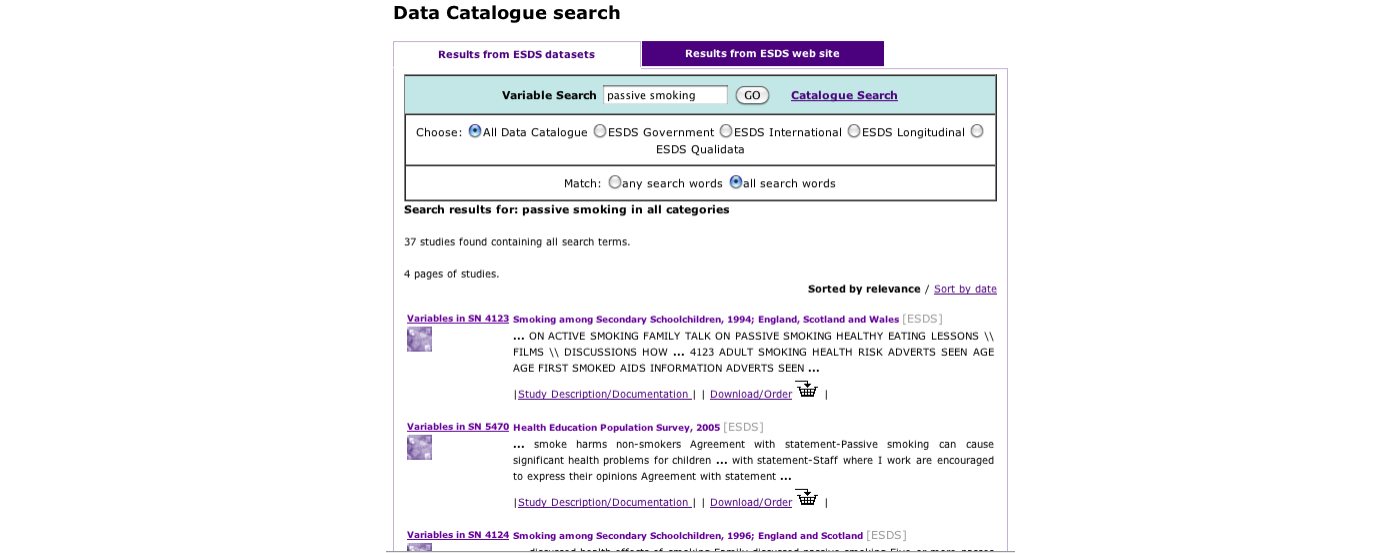
Variable Orientation -- The search box is at the top of the page, and the results are returned underneath. To view the list of variables, the user clicks the 'Variables in...' link on the left hand side of each result, which provides a list of all the variables in the dataset.

**Figure 5:**
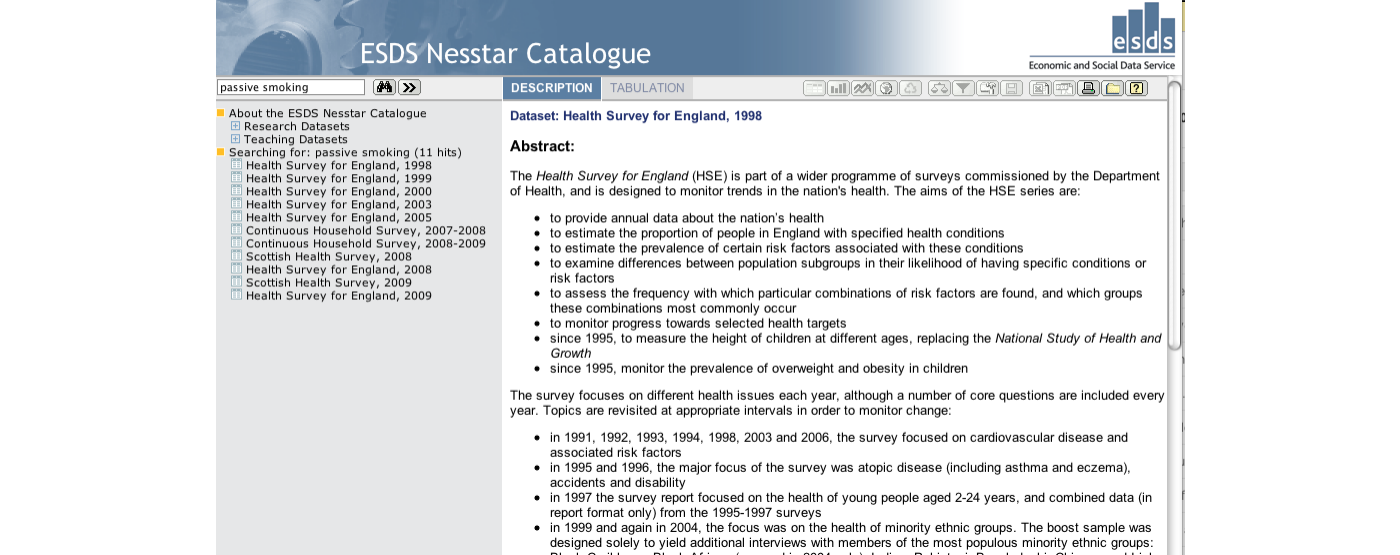
The Nesstar interface -- Surveys matching the search terms are listed in the menu on the left hand side.

The resulting surveys were listed on the *catalogue search* page (Figure 2), and searches could then be refined using the *catalogue search* form. To access the variables in the survey, the user clicked through to the “Survey Description/Documentation,” and then followed the “Variable List” link at the top of the page, which provided a list of all the variables in the dataset (Figure 3). Variable details were provided on a separate page when the user selected the name and clicked “show variable.” The variable search (Figure 4) contained a single search box, and returned surveys that contain variables matching the keywords in a list underneath. Users could click through to the survey description and view the variable list as before, or click the link on the left of the result to go straight to the list of variables. The Nesstar tool allowed users to search and browse surveys in a tree view (Figure 5), and the ESDS government variable search returned a list of variables that matched search terms just from the government surveys.

In addition to the search facilities, there were numerous routes through which users could browse the available surveys, such as the “browse by subject” and “major studies” pages. Lists of variables could then be accessed from the study description pages. The ESDS website, like many sites, was frequently edited and upgraded; the study was conducted between September 27, 2011, and November 3, 2011, a period during which there were no major changes to the functionality offered by the site.

### Comparable Web Search Interface

**Figure 6:**
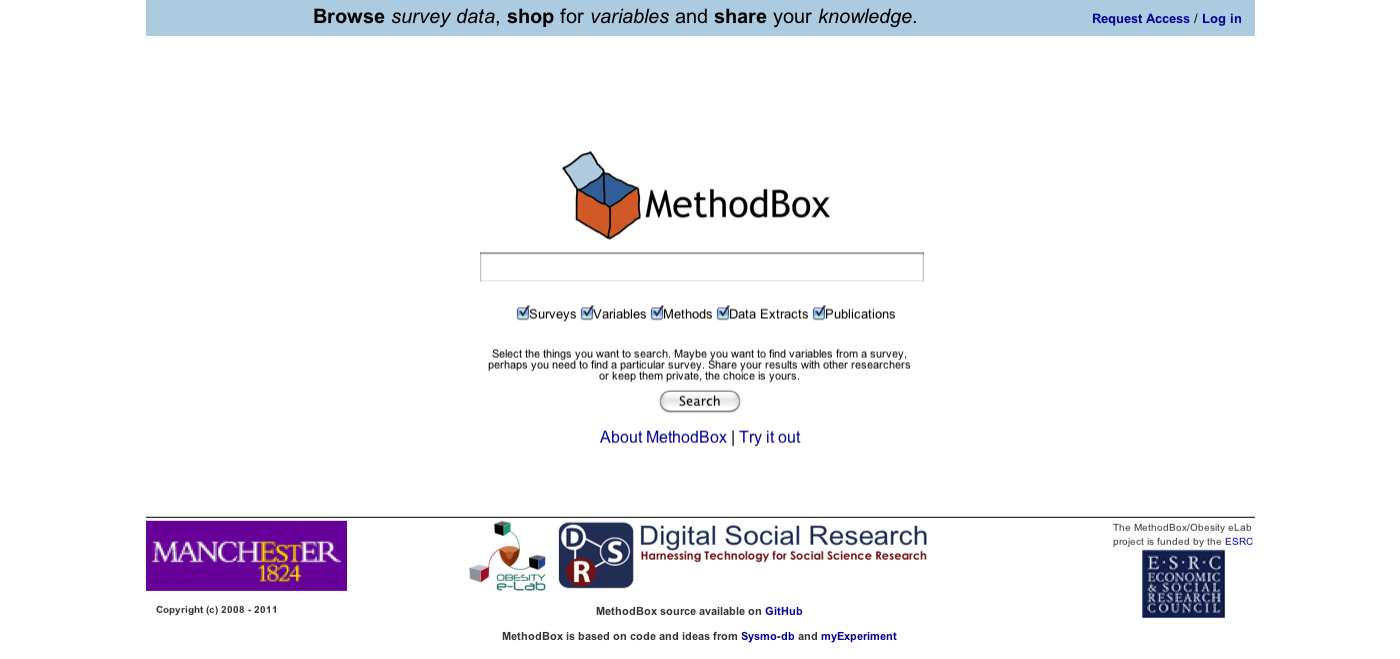
MethodBox Home -- Users type a query in the central search box, and can modify what is searched (surveys, variables etc.) using the tick boxes underneath.

The Web search interface (MethodBox) was designed to simplify the process of accessing survey data by enabling people to look for variables of interest through a straightforward “Web search” interface embedded in a scientific social network (Figure 6). Researchers typed a query in a single search box and then browsed relevant results. Variables were displayed in a table format, which could be reordered according to a number of categories. The variable description was displayed prominently, allowing users to see at a glance whether the variable was relevant to their research question. Variables of interest could be selected and then downloaded to the user’s desktop.

A clear priority identified in the requirements analysis was a fast and straightforward means of identifying variables that are relevant to a particular research question. To achieve this, MethodBox assimilates all the required information about a variable, including its name, values and metadata, using the Data Documentation Initiative (DDI) [42] XML files available through the ESDS Nesstar service, and through processing the dataset documentation with the Utopia PDF parser [43]. This process allowed MethodBox to treat variables as first-class citizens in their own right. Users could also upload their own data files and add metadata in the DDI XML format. Assets inside MethodBox were indexed using Apache Solr, allowing users to search variable names and metadata quickly and easily, as well as the surveys, data analysis scripts, data extracts (subsets of variables created by other users), publications, and user profiles also held by MethodBox.

**Figure 7:**
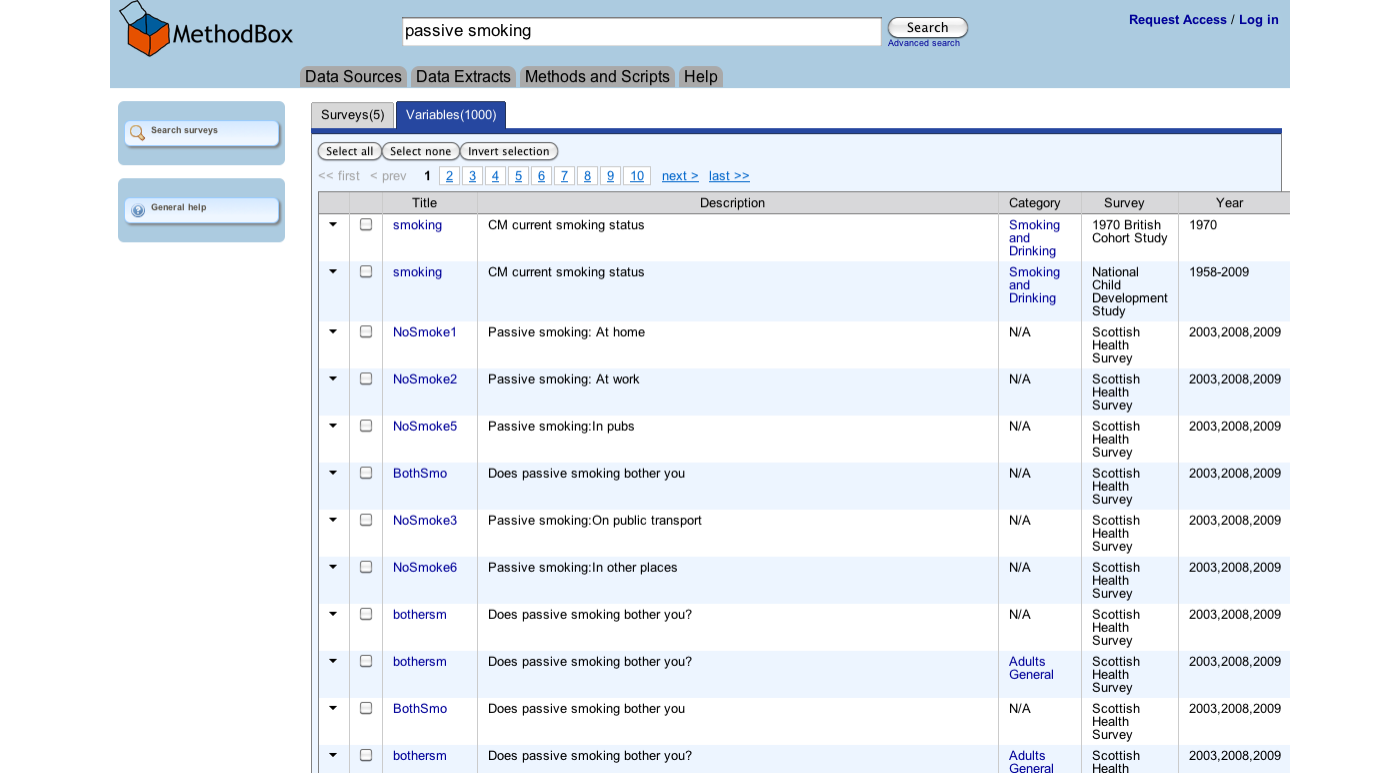
Survey Results -- Categories that contain results are displayed in separate tabs. Results are displayed in a table and ordered according to relevance, but can be sorted by clicking the table headers.

**Figure 8:**
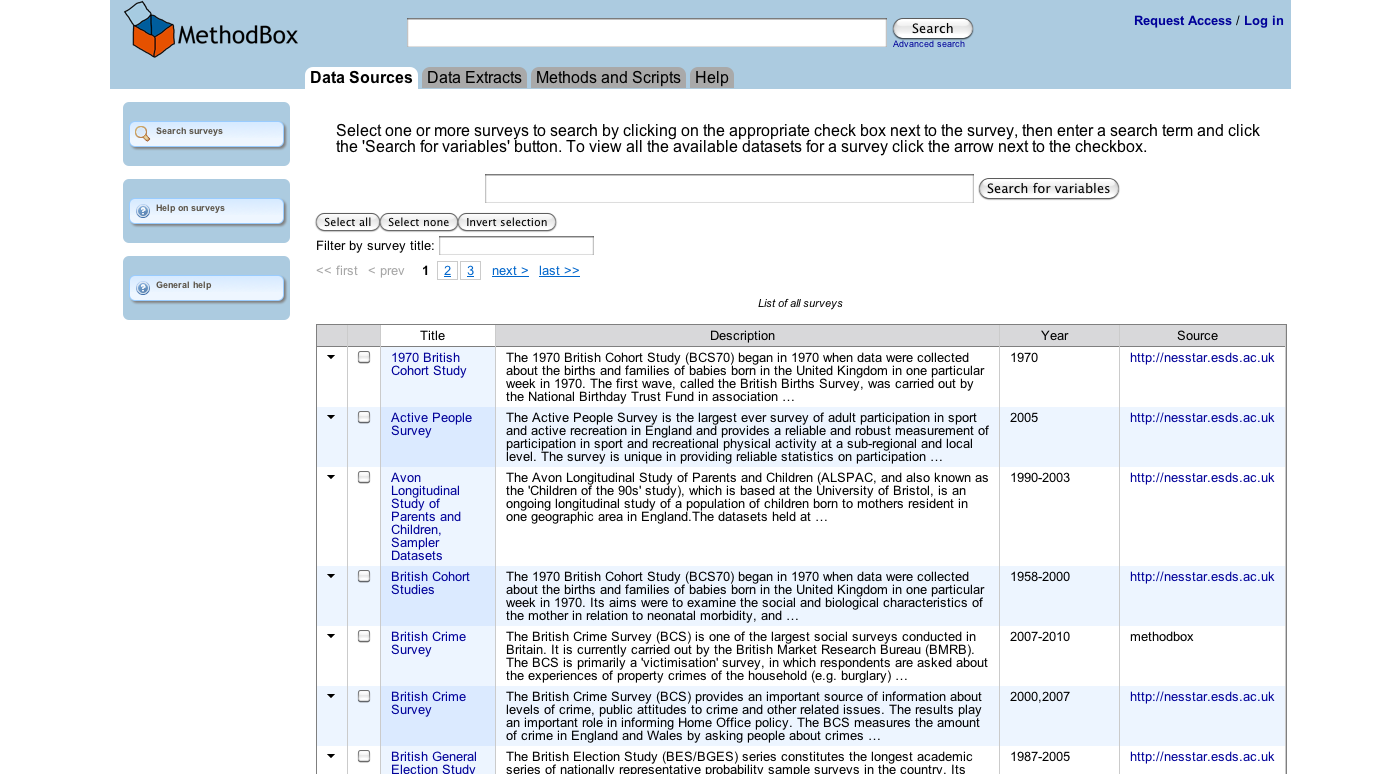
Surveys -- Users can select surveys and search them for variables.

The MethodBox user interface was designed to correspond to the common mental model of an online Web search interface: a box for entering terms, a button to run the search, and a list of results [27]. The home page consisted of a single, “Google-style” search box, with checkboxes underneath to allow users to specify what they wanted to search (see Figure 7). All categories (surveys, variables, methods, data extracts, and publications) were selected by default. Matching results were returned in a table format. Results were initially ordered according to relevance, but could be sorted, for example, by year or survey, by clicking the table headers. If there were matching results in more than one category, these were displayed in separate tabs (Figure 8). Variable details could be accessed by clicking the arrow to the left of the result, which provided them in a dropdown box, or by clicking the variable title, which showed them on a separate page. Users could also select and search a subset of surveys (Figure 8) or navigate to a complete list of the variables from a link on the survey description page. If users were logged in, they could add any number of variables to their “shopping” cart, before downloading this subset of data to their desktop as a “data extract.”

## Methods

The aim of the evaluation was to understand whether the Web search interface provided more effective, efficient, and satisfactory access to variable data stored in the UKDA than the traditional search interface.

### Hypothesis

The purpose of the study was to evaluate the Web search approach, and as such the broad hypothesis was that users would find the process of discovering variable data to be easier using the Web search interface than the traditional search interface. In particular, we hypothesized that the Web search interface would be

- More effective—users would find variables that more accurately matched their research questions, and would have more confidence in the results.
- More efficient—users would be able to find relevant variables more quickly.
- More satisfactory—users would rate the interface as more learnable, easier to use, and generally more satisfactory.

As the study provided an empirical comparison of various approaches to finding variable data, however (ie, neither website tied users to following a single “route” to data discovery), it was expected that the qualitative results in particular would help to identify the features and functionality that participants either liked or disliked and the reasons why, thus contributing to future user interface development.

### Tasks

Search tasks were developed in collaboration with the ESDS government team (ESDSG) [44] based at the University of Manchester, and were designed to reflect the kind of research questions that people may seek to answer using the survey data stored in UKDA. ESDSG designed the format for the tasks, and the details of each were decided in a discussion involving both the MethodBox and ESDSG teams. This was part of a wider initiative between medical and social research funding bodies to improve the use of social data in medical research. Participants were asked to find a variable that could be used to answer the following questions:

1. What proportion of people in Scotland believe Jesus was the son of God? *(hereafter referred to as the Belief task)*
2. What proportion of people in Wales speak Welsh fluently?

the Welsh task

1. What proportion of people in Northern Ireland have a bus link to local shops and services?

the Transport task

1. What proportion of the British population have private health insurance?

(the Health care task)

When they had found a variable that they felt gave a satisfactory answer, they were asked to say so. They were free to stop at any point if they did not think it was possible to find a variable that would answer the question.

### Evaluation

As there are numerous ways of accessing variable data using both interfaces, participants started every task on the home page of the site and were free to navigate around and use resources as they wished. Participants were given a number of focused questions, and asked to find data with which to answer them. Searching through surveys to gain a complete picture of all the data available to answer a question would be very time consuming, potentially taking days or weeks [7]. As a proxy measure participants were therefore asked to locate a single variable that provided as complete an answer as possible. All the tasks were completed using both the interfaces, providing a direct comparison between the two.

#### Experimental Design

Secondary data analysis involves researchers who are not the originators of the data. Such data are conventionally stored in archives so that a wide variety of researchers can access and reuse them. Researchers usually approach the archive with a specific question and relevant variables in mind. In the health and social sciences domain, large, complex population-based surveys are heavily reused in this way. Thus, the speed and quality of the data presentation and the facilitation of variable discovery and high task performance are critical.

To discover which type of interface best supported users undertaking secondary data analysis a repeated measures design was used. Participants searched for data to answer the same 4 questions using both interfaces. Participants were asked to approach each search afresh: that is, to look for any data with which to answer the question and choose what they thought was most useful, rather than to search only for the name of a specific variable or dataset that they knew would provide the answer. Participants completed all the tasks using one of the interfaces first, then had a short break while they answered questions about the experience they had just had, before completing the tasks in the same order using the other interface. The order of the tasks was varied according to a Latin square. The design was counter-balanced, so for every participant who completed the tasks in a given order using the traditional search interface first, another completed the tasks in the same order but using the web search interface first.

#### Participants

A total of 5 male and 15 female participants between the ages of 18 and 35 took part in the evaluation; 20 users are seen as a satisfactory sample size for understanding human interaction with a software system in this domain [45]. All participants were working or studying in the areas of social science or health science, and had some experience of secondary data analysis. A total of 11 participants had 1-year experience or less, 5 had 2-3-year experience, and 4 had 4 or more years’ experience. Participant’s previous experience with the particular tools assessed in the evaluation was very limited. Among the study participants, 1 had used both MethodBox and ESDS before a few times, 1 had used MethodBox once, and 5 had occasionally used ESDS. It should be noted that the participants who had previously used MethodBox would have encountered an earlier version with a different user interface. Other online resources participants used to look for data included the Office for National Statistics or Casweb (4 participants), Survey Question Bank (2 participants), medical databases (4 participants), European Data Centre for Work and Welfare (1 participant), and EuroStat (1 participant). A total of 5 participants worked mainly with data they had collected themselves or which came from colleagues or supervisors. Finally, our participants were between 18 and 35 years of age, as we wished to focus on digital natives and thereby make our evidence more portable to future searchers/users. However, we suggest that this focus did not adversely skew our study. While our participants were all within the 18-35-year age group, a prior work [46] showed that as familiarity increases task performance over the age ranges 20-59 harmonizes. In our context, it is unlikely that our user population would include workers much over 65 years of age. Further, even for groups over 60 years of age, no significant age-related differences in tag-based search interfaces (such as our Google-like faceted browsing) have been found [47], although differences have been found in hierarchy-based search (such as our traditional system) [47].

#### Metrics and Data Collection

Experimental sessions were audio and video recorded and participants’ eye movements and mouse movements/keystrokes were tracked using Tobii Studio Professional software. Task completion times and correctness scores were calculated, and participants’ behavior and comments during the sessions were documented and analyzed. Eye-tracking data were used to provide insight into situations that could not be understood using the other measures alone; for example, to determine whether a participant was ignoring a matching variable that had appeared, or had not seen it.

After each task, participants were asked to rate, on a scale of 1-7 (with 1 being “not at all” and 7 being “very”) how confident they were that they had found a satisfactory answer, and how easy they found it to obtain their answer. After they had completed all the 4 tasks using the single interface, they were asked to rate, on a scale of 1-7 (with 1 being “not at all” and 7 being “very”), how easy they found it to learn how to use the interface, how easy it was to find data using the interface, and their overall satisfaction with the interface. They were also asked to state what they liked and did not like about the interface.

After they had completed the tasks, participants were asked to state which interface they preferred using for finding variable data, and to provide a reason for this.

#### Set Up and Procedure

The Web search interface provided an alternative view on the data stored in the UKDA, but did not provide access to the same amount of data as the traditional search interface (eg, census data were not available through the web search interface). The questions were designed, so relevant answers could be found in the UK government surveys that can be accessed through both the web search interface and the traditional search interface. Both sites were checked to confirm that at least one variable (the same in each case) containing all the information required to answer each question could be found. Because both sites were live and independently updated, it is possible that they contained other, potentially different matches.

Participants completed a consent form and an entry questionnaire about their previous experience of finding quantitative survey data for secondary analysis. They were then asked to consult the appropriate help documentation for the first interface. For the traditional search interface, this involved reading the online “Help on Searching the Data Catalogue” [44] document (participants were directed in particular to the section on searching for variables) and in the case of the web search interface, reading the “About” [37] page and watching the help video. Participants completed the 4 search tasks using the first interface, providing confidence and ease-of-use ratings after each task, and learnability, overall ease of use, and satisfaction ratings when they had completed all the tasks. They then repeated this process using the second interface. Finally, they stated their preference for either the Web search or traditional search interface, and provided a reason for this.

#### Statistical Analysis

The usability metrics were analyzed using a task × interface repeated measures generalized linear model (GLM) procedure and the Greenhouse-Geisser correction was made when looking at task effects. For the overall (all tasks) scores of each aspect of usability, the differences between interfaces were summarized with a confidence interval for the mean difference and a paired Student *t* test *P* value. The distributions of some metrics were a little asymmetrical, and therefore, sensitivity analyses were performed using alternative permutative nonparametric methods, which gave almost identical results. We present the main effects of the parametric analyses with a 95% confidence interval unless otherwise stated. We use a 5% statistical significance level and 0.1% high significance level. Post hoc pairwise comparisons were adjusted for multiple testing. Calculations were performed in StatsDirect 3.0 and SPSS 19.

## Results

### Observations

When using the Web search interface participants started each task on the home page containing the main search box. Just over half of the participants used the checkboxes underneath the search box at least once to restrict the search to variables (6 participants) or variables and surveys (7 participants).

Participants looked for variables by entering terms into either the main search box (in the center of the home page and at the top of the page throughout the rest of the site) or the variable search box on the survey tab. A total of 13 participants chose at least one variable after only a single search; the rest of the time participants performed two or more searches before they found an answer they were happy with. A total of 5 participants chose to search within particular surveys at least once; 3 participants reordered the results table at least once and 7 clicked the “show all variables” link for a particular survey, although only 3 ended up choosing a variable from this list, with the rest returning to the search facility. Half of the participants looked only at the first page of results before either choosing a variable or searching again; the other half looked beyond the first page for 1 task (4 participants), 2 tasks (5 participants), or 3 tasks (1 participant). Observation of participants’ eye movements showed that they made a decision about whether or not to view a variable’s details primarily by glancing at the “description” column of the results table.

In a number of instances, participants found a variable that answered the question quickly, but did not choose it straightaway. A total of 7 participants hesitated to choose a variable because there were several in the results that would answer the question. In 5 other cases, the eye-tracking data showed that participants saw a correct answer in the first set of search results, but spent some time looking around the page or other parts of the site before choosing the variable as their answer.

A total of 3 participants failed to find (in their opinion) a satisfactory variable in 1 task using the web search interface, and 1 failed in 2 tasks.

When using the traditional search interface, 4 participants failed to complete 1 task, 2 failed to complete 2 tasks, 1 failed to complete 3 tasks, and 4 did not complete any.

There was a much greater variety in the way that participants used the traditional search interface. A total of 4 participants used only the variable search and 2 used only the catalogue search; the remaining participants used a combination of the simple search on the home page (11 participants), the variable search (14 participants), and the catalogue search (13 participants). As much as 11 participants used more than 1 search facility within the same task and 14 used different search facilities across different tasks; 7 participants tried Nesstar, but only 1 participant found a satisfactory variable using this tool. A total of 10 participants chose to use the browsing facilities (such as the “browse by subject” page) to access study descriptions, in addition to searching. None of the participants accessed the government variable search, possibly because there were no prominent links to it from the home page or help documentation.

A total of 7 participants consulted the help documentation when using the traditional search interface, compared with 2 when using the Web search interface; 6 participants used the browser’s “Ctrl + F” command at some point to locate text within a page with the traditional search interface, whereas only 2 participants used this approach with the Web search interface.

A total of 13 participants chose to look beyond the first page of the results following a search. Because variables had to be located within a list of all the variables in the dataset, it was typical for participants to spend a long time scrolling before they reached the answer.

### Performance

The performance measures were the correctness of the results and the time taken to complete the task.

Because the traditional search interface provides access to a greater volume of data (and the Web search interface a subset of these data), it is possible that it may contain more relevant variables, increasing the chance that participants may find a correct answer. However, it is also possible that this may have a negative impact on task completion times, as the larger data collection may take longer to search.

**Table 1:** Mean (SD) correctness scores for each task.

Task	Web search interface	Traditional search interface
Belief	2.40 (0.88)	1.05 (0.94)
Welsh	2.45 (0.76)	1.65 (1.09)
Transport	1.95 (1.00)	0.55 (0.94)
Health care	2.70 (0.92)	0.85 (1.23)
Overall	2.38 (0.89)	1.03 (1.05)

Correctness was scored out of 3. If participants found any variable containing all the required information, a score of 3 was given; finding a variable that contained most of the information received a score of 2; finding a variable that contained some of the information received a score of 1; failing to find a relevant variable received a score of 0. Participants were not asked to consider year as part of the search criteria. An investigator from each of the MethodBox and ESDSG teams rated correctness, and reached a consensus about the appropriate value where there was disagreement.

**Table 2:** Mean (SD) completion times in seconds for each task.

Task	Web search interface	Traditional search interface
Belief	159.1 (110.5)	243.5 (159.1)
Welsh	143.9 (80.6)	202.8 (148.9)
Transport	208.0 (161.5)	309.8 (153.7)
Health care	163.0 (100.1)	313.8 (135.9)
Overall	168.5 (113.2)	267.5 (149.4)

Table 1 shows the mean correctness values for each task. A task × interface repeated measures GLM procedure shows a main effect of interface, indicating that answers found through the Web search interface were more likely to be correct (*F*_1,19_=37.3, *P*<.001) and a main effect of task (*F*_3,57_=6.3, *P*<.001), with post hoc pairwise comparisons showing that participants obtained significantly lower scores in the transport task than any of the others. There was also a task × interface interaction effect (*F*_3,57_=3.3, *P*=.028), which reflects the fact that while correctness scores were lowest for both interfaces in the transport task, scores for the health care task were the second lowest using the traditional search interface, but highest using the search engine interface.

Observations of the search process show that while participants encountered, on aggregate, more than 5 variables that would provide a correct answer in the Welsh task, and more than 20 variables that would answer the health care question, in the case of the belief and transport tasks, all participants who achieved a score of 3 chose the same, single variable, which was the only correct answer to appear during any search. The correctness results for the Web search interface, which showed participants achieved the highest scores for the health care task, followed by the Welsh, belief, and finally transport tasks, broadly reflect this fact. When using the traditional search interface, however, participants obtained the second lowest score for the health care insurance task, and therefore, the correctness scores do not appear to vary simply as a function of the number of available answers. In fact, the more important factor appears to be the position of the answer in the variable list; by contrast, the answers chosen in the Welsh task were right at the top, the variables relating to health care were much further down, and many participants simply gave up on the dataset before they got to them.

Table 2 shows the mean completion times for each task. A task × interface repeated measures GLM procedure shows that participants completed the task significantly faster using the Web search interface (*F*_1,19_=18.0, *P*<.001). There was also a main effect of task (*F*_2,38_=4.1, *P*=.025). Post hoc pairwise comparisons indicate that this was due to the transport and health care tasks taking significantly longer time to complete than the Welsh task.

A task order × interface repeated measures GLM procedure was conducted to check for task order effects. There was a main effect of interface, showing that people completed the tasks significantly faster using the Web search interface (*F*_1,16_=8.6, *P*=.01), but order did not have a significant effect at the 5% level (*F*_3,48_=2.2, *P*=.1), and there was no interaction effect (*F*_3,48_=0.6, *P*=.6), indicating that there was no significant difference in the rate at which participants learned to use the interfaces.

**Table 3:** Mean (SD) ratings for overall interface learnability, ease of use, and satisfaction.

Variable	Web search interface	Traditional search interface
Learnability	5.55 (0.94)	4.05 (1.23)
Ease of use	5.88 (0.76)	3.70 (1.80)
Satisfaction	5.78 (0.87)	3.15 (1.66)

### Overall Ratings

After participants had completed all the tasks using an interface, they were asked to rate on a scale of 1-7 its overall learnability, its overall ease of use, and their overall satisfaction with it (Table 3). Paired comparisons showed that the Web search interface received significantly higher ratings than the traditional search interface for overall learnability (*P*=.002, 95% CI [0.6-2.4]), ease of use (*P*<.001, 95% CI [1.2-3.2]), and satisfaction (*P*<.001, 95% CI [1.8-3.5]). It is interesting to note that whereas there is only a 1.5-point difference between the traditional search interface and the Web search interface for learnability, for ease of use this rises to 2.2 points, and for overall satisfaction it rises to 2.6 points.

### Confidence and Ease-of-Use Ratings for each Task

After completing each task, participants rated on a scale of 1-7 how confident they were that the variable they had found answered the question, and how easy it was to find the answer.

**Table 4:** Mean (SD) confidence ratings for each task.

Task	Web search interface	Traditional search interface
Belief	5.65 (1.90)	2.73 (2.47)
Welsh	4.78 (2.00)	4.30 (2.62)
Bus	4.85 (1.87)	1.78 (2.26)
Health care	4.63 (1.99)	2.25 (2.00)
Overall	4.98 (2.77)	1.94 (2.34)

Table 4 shows the mean confidence ratings for each task. A task × interface repeated measures GLM procedure indicates that participants were significantly more confident about their answers when using the Web search interface (*F*_1,19_=18.8, *P*<.001). Post hoc pairwise comparisons show that participants were significantly more confident about their answers in the Welsh task than in the transport or health care tasks, and significantly less confident about their answers in the health care task than in the belief task (*F*_2,38_=4.7, *P*=.015). A task × interface interaction effect (*F*_3,57_=4.4, *P*<.01) indicates that the confidence rating varied according to the interface: in the Welsh task, participants had a similar level of confidence in their answer, but for all other tasks it was much higher when using the Web search interface.

Table 5 shows the mean ease-of-use ratings for each task. A task × interface repeated measures GLM procedure shows that participants found the Web search interface significantly easier to use (*F*_1,19_=14.0, *P*<.001). There was no significant effect of task (*F*_3,57_=2.2, *P*=.1).

**Table 5:** Ratings: mean (SD) ease of use ratings for each task.

Task	Web search interface	Traditional search interface
Belief	5.08 (1.78)	2.95 (2.39)
Welsh	4.85 (1.87)	3.88 (2.42)
Bus	5.03 (1.92)	1.90 (2.31)
Health care	4.70 (2.11)	2.68 (2.19)
Overall	4.92 (1.92)	2.85 (2.33)

### Qualitative Feedback

Participants were asked for qualitative feedback at two points: after they had completed all the tasks with an interface, they were asked to say what they liked and disliked about it; and at the end of the study, they were asked which interface they preferred and why. In addition, participants made occasional remarks about the interfaces while they were completing the tasks; these comments are also included in the analysis that follows.

#### Completed Tasks and Remarks

A total of 18 participants said they preferred using the Web search interface to search for and access variables; 9 participants stated this was because it was more user-friendly or easier to use. According to a participant

*It’s easier to find variables and the information is clearer. In [the Traditional Search interface], the information is in another file or in another link. [In the Web Search interface] it’s just there so I can see it easily.* [Participant Number 18, Female].

A total of 7 participants mentioned that the search process was quicker when using the Web search interface

It’s so much faster. You’d just get so annoyed with [the traditional search interface] because of the amount of effort.

Participant Number 15, Female

When I searched for something, I was able to see whether the results were relevant more immediately than with [the traditional search interface].

Participant Number 3, Female

When all the information came up I was able to scan it quickly and see, well this one is relevant and this one isn’t.

Participant Number 14, Female

A total of 4 participants described the Web search interface as more intuitive

The format of the site means it’s more intuitive how to get around it, how to find stuff.

Participant Number 12, Male

One of the participants provided the following reason for liking the Web search interface *I could find what I was looking for.* [Participant Number 2, Female]

A total of 7 participants commented on the simplicity of the interface.

It’s easy because you can just search one comprehensive way rather than spending time debating which method you’re going to use to actually look for your data

Participant Number 9, Female

One participant said this could undermine confidence in the interface, however

I definitely preferred [the Web search interface], but I know this might sound weird but because it was so easy you worry that what you’ve done is not right, or it’s not reliable

Participant Number 17, Male

P7F said that although she preferred the output of the search process in the Web search interface, she preferred using the catalogue search of the traditional search interface to specify search terms:

[the traditional search interface] felt a bit more open, whereas this [the Web search interface home page]—everything’s hidden behind it. I felt happier with searching with the traditional search interface.

Participant Number 7, Female

Another participant, *Participant Number 20, Female*, who said she preferred using the traditional search interface, also cited the catalogue search facility as the reason, saying “it allows you to provide more details and filter the search.”

#### Postsystem Interview

In the postsystem interview, the Web search interface received 35 positive and 17 negative comments, whereas the traditional search interface received 12 positive and 25 negative comments. A total of 6 participants said that they found the Web search interface easy to use, and 6 commented on its speed and simplicity:

*It’s faster than [the traditional search] interface—you get the same results with fewer clicks* [Participant Number 13, Male].

Two described it as “user-friendly”:

*[the Web search interface] is probably more user-friendly because [the Web search interface] is pretty much like the Google one, so the user may be more familiar with this kind of searching method.* [Participant Number 11, Male].

An additional 2 participants compared the search facility favorably with Google and 4 others liked the simple, familiar format.

It just seemed so easy, normal—[an] Internet search engine but with a different purpose

Participant Number 17, Male

However, some disliked the simplicity of the search box:

I didn’t really like the fact that [the main search page] was it. I couldn’t automatically do a date or a region filter

Participant Number 7, Female

As much as 9 participants said they liked the format of the results. According to one participant,

The returns I got were more helpful than with [the traditional search interface]...I think [the Web search interface] has a better grasp of what researchers actually want, so I liked the fact that once you’d got your search returns it said what exactly was the wording of the question, and what category that came under, because sometimes a question will have a different meaning if it’s asked under demographics, or asked under some other category, so you might just look and say, “that’s not relevant”

Participant Number 7, Female

A total of 4 participants liked the fact that you could search for, or within, particular surveys and one said she liked the help video.

The negative comments about the Web search interface in the interview were mainly related to the description of particular variables. A total of 4 participants commented that values for some variables did not seem to be available:

*I disliked the fact that some of the variables did not seem to have information in—that confused me. I don’t know whether that means they’re searching datasets they don’t have information for? That could be made clearer.* [Participant Number 3, Female].

Of the study participants, 2 found the appearance of many variables with the same title confusing, and one felt that the wording of some variable titles was unclear

*Some of the questions said things like, “Bus stop, feel, don’t know”...I don’t know what that question means* [Participant Number 8, Female].

However, P8F did recognize this as a potential problem with the survey, rather than with the interface. Also, 2 participants commented that it was not always clear which year variables applied to, and one wondered about the geographical location of the study, which was not apparent just from looking at the variable description.

It was suggested by 2 participants that the Web search interface returned too many variables in the search results, although

*that can be managed if you sort them according to which survey they are taken from etc.* [Participant Number 13, Male].

One of the participants lacked confidence in the search due to the simplicity of the interface

It’s a little less transparent as to what’s in the box...I think I’m doing the right thing but I’m not sure.

Participant Number 7, Male

There were also 2 other participants who found it hard to find the keywords to bring up the required data. One participant commented on the fact the “back” button did not work properly and another did not like the format of the help documentation.

When asked what they liked about the traditional search interface, two key areas came up. A total of 4 participants found the extensive help documentation useful and 5 liked the options provided for filtering results:

*It’s easier to have a general idea of categorizing topics and areas...you’re more likely to exclude something that is not what you want, or include what you want.* [Participant Number 11, Male].

Another participant commented,

*It looked a lot more professional than [the Web Search interface]. I got the impression it had access to a lot more data.* [Participant Number 2, Female].

One participant also mentioned that she found the Nesstar tool helpful (P5F).

When asked what they disliked about the traditional search interface, 3 participants said they found it complicated or hard to use, one described it as less intuitive than the Web search interface and one said it was slower. As much as 4 participants said they found it difficult to get any useful results at all and 8 said their searches returned too much information. One participant mentioned,

You felt that what you got out was quite vague, or not to the point of what you wanted. It just seemed to come up with all sorts of stuff that was completely irrelevant and just wasn’t very helpful. Because it would bring up so many items you couldn’t really go through them.

Participant Number 17, Male

Another suggested,

I think it would have been really useful, if they brought up say 200 datasets, if the variables you were actually looking for were highlighted in the small amount of text you’ve got underneath the heading, because then you can make a judgement.

Participant Number 3, Female

A total of 5 participants complained about the fact that the results did not give you direct access to the variable data:

I thought I’d worked it out then realized I hadn’t. It wasn’t easy going from one step to another—it was kind of frustrating.

Participant Number 8, Female

According to another participant,

*There’s too much supplementary material before you knew whether that was what you were really looking for or not.* [Participant Number 19, Female].

One participant complained that there was no option for sorting the results (Participant Number 13, Male). Another said that it was odd that the variables search was so limited, when compared with the catalogue search:

it just gave you a single box...it didn’t give you the ability to search by region and keywords

Participant Number 7, Female

One participant noted that it was difficult to choose how to look for data: *I’d start searching one way, then I’d think, maybe I should search that way...* [Participant Number 9, Female]

As participants were completing the tasks, they were more likely to make negative comments than positive ones. The Web search interface received 6 positive comments, 4 of which stated that the interface was easy to use. P19F commented on the fact that she had successfully found a variable, and P7F said that she found it helpful to be able to see what a variable contained “without having to go into it.” The only 2 positive comments to be made about the traditional search interface also came from P7F, who said that the catalogue search seemed more efficient than the variable search, and after the second task,

I found the searching slightly easier this time because I’d got the hang of it.

Participant Number 7, Female

Of the 13 negative comments made about the Web search interface while people were completing the tasks, 5 were due to bugs or errors, including the help video being of an inadequate resolution (1 participant), the back button not working correctly (1 participant), and terms in quotation marks that contained white space not being found (3 participants). Besides, 2 participants commented on the lack of an advanced search facility, of the type provided by the traditional search interface, and 2 disliked the fact that a search did not match only complete words (eg, a search for “bus” would return results with “transport” in the variable title). A total of 3 participants commented on the presence of what looked like ghost variables in the results:

It’s confusing me now. I found what I think are the ones I was looking at before [in the traditional search interface] but when I actually click on it, it’s saying that there’s no source, no metadata, no value, so I don’t really know whether I have found it. I still found the data easily but I’ve got no idea.

Participant Number 3, Female

Of the 16 negative comments participants made while they were using the traditional search interface, 4 were people expressing their dislike for or frustration with the system: *I am actually just getting really annoyed now* [Participant Number 15, Female].

A total of 5 participants commented that it was taking too long to find a variable, and 3 said they were confused or finding the process too difficult. Some participants (n=4) also complained about the format of the search results, including the fact that the variable search returned surveys, rather than taking you directly to the relevant variables (2 participants), and the fact that certain survey years did not seem to appear in the results when they were known to exist (2 participants). P7F also complained about the fact that you could not search within a survey for variables, saying that the variable lists for the datasets were “an awful lot to try and read through.”

## Discussion

This study has shown that the functionality provided by the Web search interface was preferred to that of the traditional search interface for finding variables in research data archives. Participants were more likely to find a variable that correctly answered the question posed by a task. In addition, they were able to do this more quickly and had more confidence in the results. They found the Web search interface easier to use, and were more satisfied with the overall experience it provided.

We now consider these findings in the context of the wider evidence base: specifically, the merits of leveraging the Web search approach to help users find variables, considering it within the context of HCIR literature.

*Query formulation and query reformulation* strive to put control of selection and interpretation of results in the user’s hands. This is accomplished by allowing the user to quickly formulate and reformulate the query as their understanding of the search domain increases based on the results returned. The Web search interface appears to support this well:

*It was simple to use, cause I just used keywords, and I used the same keywords in the other thing and it couldn’t find it...* [Participant Number 14, Female]; and

It’s a quick way of finding what variables there are...if you were just looking at say pay, and you just wanted to look at income...I worked on a project looking at minimum wages and things like that, so we mainly use EUROSTAT, but if you could search for something...[the Web search interface] would have been really useful for that kind of thing.

Participant Number 9, Female

These examples indicate the broader feeling that traditional search interfaces require a more precise conceptualization of what is required and available for search. By contrast, the Web search interface relies far less on the users’ knowledge of the base data and so is better for variable search.

*Browsing* is generally considered to involve virtually no planning, preparation, or focus. This kind of interaction is common in Web search and is related to query formulation and reformulation, requiring less initial knowledge of the data available. Participants’ comments suggest the interface supported this activity:

It seemed to be an easier step between the search term and the list of variables

Participant Number 5, Female

When all the information came up I was able to scan it quickly, and see well this one is relevant and this one wasn’t

Participant Number 14, Female

Browsing for relevance appears to be key to the variable data discovery performed in the study.

*Faceted search and navigation* enables users to group and interact with information hierarchically, and is becoming both expected and critically important for refining search results. Participants commented that being able to limit results in the Web search interface was useful:

I like the different layers of options, so if I search for some different variables and some surveys clearly won’t be at all relevant I can select them out.

Participant Number 3, Female

I like that you could click to look for particular surveys or particular variables. So for example if you’re looking for something in Northern Ireland (NI), I could choose NI surveys and exclude everything else so I don’t get swamped with variables, because if you do type in just one thing you get an awful lot of answers coming back and you could get quite lost, so that seemed good.

Participant Number 8, Female

*Surrogates* are the titles and abstracts for documents; thumbnails for Web pages, etc, which can be seen interspersed within the search results of modern Web search interfaces. Indeed, amalgamations of surrogates can be seen in many Google searches with documents and information being displayed from Wikipedia and from more general image searches. Surrogates in our Web search interface were limited to the title of the survey. One participant commented that

It helps to know where [the variable] is from.

Participant Number 4, Male

*Relevance feedback* modifies an existing query based on available user-based relevance judgments for previously retrieved documents. One participant commented that

in the title of the [variables] they have a lot of information there so it is easy to know when you have found it.

Participant Number 18, Female

*Summarization, analytics, and visual presentation* can enable users to better digest the query result, and formulate queries in a familiar interface. Indeed, we received many positive comments on this part of the interaction design: One noted that

*Pretty easy [to learn how to use]—it’s like a basic search engine* and *I like the layout; everyone knows how to use Google so everyone can find the variable they want.* [Participant Number 2, Female]

Another participant commented,

I liked it. It was like a Google search really. It’s very familiar—it’s like Google search. You just put in all the search terms and it gives up the list, rather than having to go through all the different stages of digging through the literature.

Participant Number 15, Female

One felt that

It just seemed so easy, normal—[an] Internet search engine, but with a different purpose.

Participant Number 17, Male

### A “Web Search Interface” for Research Variables

The Web Search interface was designed to simplify the process of finding and extracting variables for secondary research. Providing a familiar look, feel, and functionality was a key goal of the design process. It was designed as a Web search engine—set within a scientific social network where users can share methods for relating, extracting, and manipulating data—to take advantage of the fact that the most familiar experience of finding information for its target users will have come from the Web.

Participants were very positive about this approach, stating explicitly that they liked the fact that it resembled a familiar Web search engine; other participants commented on how quick, user-friendly, simple, and intuitive it felt. Although both interfaces provided a single-box entry system, only the Web search interface provided users with the look, feel, and functionality of a search engine like Google.

It was not only the simplicity of the landing page that was behind the Web search interface’s success, but also the format of the results. With the exception of the more detailed catalogue search, the facilities for entering search terms—a single box—were very similar for both interfaces. Whereas the Web search interface presented users with a list of matching variables, the traditional search interface provided a list of surveys, with at least one further click, and possibly some scrolling, required to reach the variable of interest. For some participants, this simply made the task more time consuming. For others, it made it impossible: users expected to see what they were searching for straightaway and when they could not they assumed that something had gone wrong, either with the search process itself or the way they were using it. The traditional search interface was designed for the retrieval of variable data the user already knew to exist; only the Web search interface truly supported the discovery of new data.

This tells us that presenting relevant results immediately after a search is very important: if a user is searching for a variable, and gets back a survey or dataset, they find this confusing. What else can we learn about the format that the results should take? It is not possible to determine from this study whether the Web search interface takes the optimal approach to formatting variable search results, but there is evidence that it uses at least an adequate one, as participants were able to find a variable that mostly or completely matched the search criteria the majority of the time. In fact, participants’ comments indicated that they were very happy with the presentation of the results. The majority said that they liked it, and those who specified why focused on the fact that the relevance (or otherwise) of the variable could be seen at a glance. The eye-tracking data show that participants made the decision about variable relevance primarily by looking at the “description” column of the results, and therefore, providing a summary of what the variable contains, and not just its title, appears to be important.

### Improving the Web Search Interface

Although participants preferred the Web search interface, it did receive some negative feedback. In several instances this was the result of a bug (eg, the back button not working properly), but it also resulted from the fact that incomplete or seemingly inaccurate variable data were sometimes returned in the results. It is likely that these problems were caused not by an error in the interface itself, but by the original format of the data in question, which was confusing. Nevertheless, ensuring that users fully understand the results that are returned to them, and why they appear as they do, remains a usability challenge for systems providing access to this type of data.

As practicing health science professionals our users have some experience using the traditional interface, but they do not have many years’ experience. We also see that they will be more familiar with a Web search interface as this is what they use most days of the week and often multiple times a day. Conforming to this model should be our primary goal as there is a much more familiarity and there is little refamiliarization required, as there is with a traditional but seldom used, interface.

### Study Limitations

As the study was conducted with live websites, it was not completely controlled. The traditional search interface searched a larger data catalogue than the Web search interface, and although this meant that it potentially produced a greater number of correct answers, this did not appear to provide any advantage from the perspective of correctness scores. The time it took a search to complete varied considerably for both interfaces, from less than a second to (occasionally) more than 10. Search times were not deducted from task completion times for a number of reasons: it was not always possible to determine how long a search took (participants often opened a new window over the top to continue with the task when there was a delay); the time to access the server is a property of the system, and as such it may not be appropriate to ignore it; searches rarely took longer than a few seconds. Nevertheless, it should be noted that the task completion times recorded for the Web search interface may rise if the proportion of the data catalogue it provides access to and/or the number of people using it increases.

A second limitation is that the study was conducted with relatively inexperienced researchers. This particular group was used because they are known to have difficulties with data discovery. There may be circumstances where the facilities provided in the traditional search interface are preferable to researchers with more experience, or those currently provided in the Web search interface are not sophisticated enough. Because of the ubiquity of Web search and our participants’ constant exposure to this, we might assume that they would be better at Web search.

A third limitation of the study was that it compared only two tools for accessing variable data. This study is one of the first to investigate user preferences for finding and accessing variable data; further work considering other tools or methods is undoubtedly necessary. It would also be useful to examine researchers’ preferences in longitudinal, naturalistic settings, as well as controlled, laboratory-based studies.

### Study Impact

**Figure 9:**
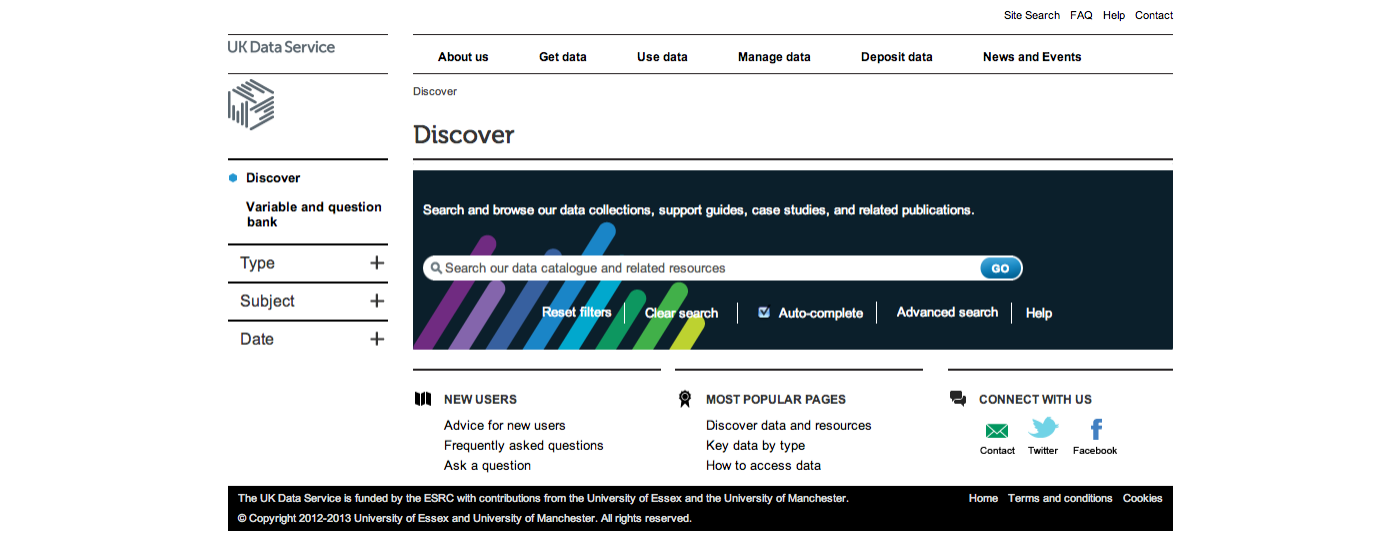
New UKDA Interface -- 'Discover' adopting the Web Search Interface and including: Faceted Search (at left); Query Reformulation (at centre); and Browsing (at centre) features.

Previously, researchers working with survey data from the UKDA found it difficult to discover relevant variables for analysis. These difficulties were compounded as single-domain researchers became cross-domain data scientists. To address these difficulties, a Web search interface (MethodBox) was designed as an alternative front end to the archive, enabling users to search through multiple sets of data, supporting documentation and user-contributed metadata in a single process. Since this study, the main UKDA search interface has been significantly overhauled to take account of the findings (Figure 9) and the new-way data are used and searched for.

**Figure 10:**
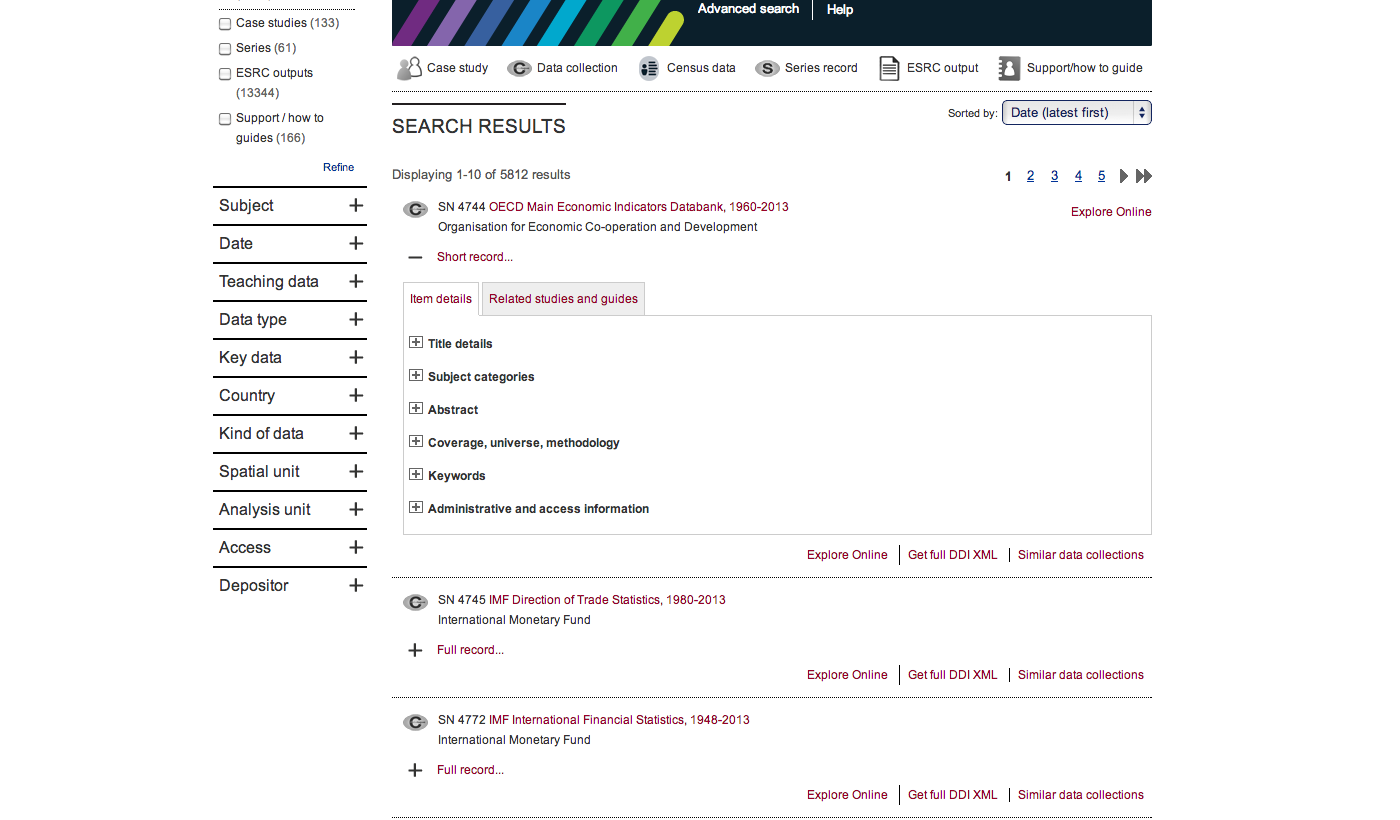
Discover Results -- Search results adopting the Web Search Interface and including: Surrogates (at centre as part of 'Full Record' detail); Relevance Feedback (at top-right as 'Sorted by:'); Summarisation (at centre with each result); Analytics and Visual Presentation (at top-left) features.

The new “Discover” Variable and Question Bank interface adopted the Web search interface paradigm described by HCIR and this was shown to be effective for variable search in this study. The interface implements all those features found to be useful to researchers, including faceted search; query reformulation; browsing; surrogates; relevance feedback; summarization, analytics, and visual presentation (Figure 10). The main aim of the study was to empirically support the anecdotal supposition that data scientists share more in common with the “Google Generation” than with their single-domain, single-tool forebears. We studied this with real applications built directly because of this anecdotal supposition; the evaluation of the MethodBox Web search interface provided empirical support for this supposition, which has implications for scientific data search and selection more generally. We have shown that users find the Web search engine approach intuitive and that it helps them to assemble relevant variable data for research. The findings apply not only to MethodBox but also to similar systems that support the need to search for variable data.

The implications of this study for the process of secondary data analysis are substantial. Many researchers, particularly inexperienced ones, or cross disciplinarians, struggle to identify the datasets and variables they should be using to answer a research question. By enabling users to quickly search a data archive at the level of recorded factors/variables, information systems can help users to focus on research rather than on the process of negotiating archives or documents.

A straightforward means of searching provides a greater opportunity for finding relevant factors/variables that the researcher was not previously aware of. This may reduce “investigator bias,” whereby research artificially focuses on familiar datasets, but not necessarily those most relevant to the hypothesis.

The simple provision of a Web search interface will not ultimately eliminate the need for researchers to “get to know” a dataset in detail, but it could make the process of data discovery quicker, easier, and far less intimidating. In turn, this may generate “digital crumbs” of metadata about the relationships between variables, users, and research processes. Such metadata may eventually support crowd-sourced secondary research.

### Panton Principles and the Science Code Manifesto

Science is based on building on, reusing, and openly criticizing the published body of scientific knowledge. For science to effectively function, and for society to reap the full benefits from scientific endeavors, it is crucial that scientific data be made open. In this case, we support the “Panton Principles” [48]. We further assert that “Code is Method” and likewise support the Science Code Manifesto [49]. In this case, we would like to invite you to access our data and code, and question our analysis and interpretation of that data via the full dataset, experimental protocols, and methodologies [50].

## Abbreviations

DDI: Data Documentation Initiative
ESDS: Economic and Social Data Service
ESDSG: ESDS government team
EU: European Union
GLM: generalized linear model
HCIR: Human-Computer Information Retrieval
IPCSR: Inter-University Consortium for Political and Social Research
UKDA: UK Data Archive
